# Supplementary material for: Challenges and opportunities for statistical power and biomarker identification arising from rhythmic variation in proteomics
Source: NPJ Biol Timing Sleep. 2025 Jan 25;2:3. doi: 10.1038/s44323-024-00020-2 (PMC11762406; doi:10.1038/s44323-024-00020-2)
Supplement: Supplementary file 1 — Supplementary Material [file 44323_2024_20_MOESM1_ESM.docx]

**Rhythmic variation in proteomics: challenges and opportunities for statistical power and biomarker identification**

**Supplementary Material**

**Table S1**: Proteins (genes) with 12 hour rhythm analysed by STRING, pathway outputs

| **GO term** | **Description** | **Count in network** | **Strength** | **FDR p-value** |
| --- | --- | --- | --- | --- |
| GO:0030195 | Negative regulation of blood coagulation | 3 of 46 | 2.33 | 0.0045 |
| GO:0042730 | Fibrinolysis | 2 of 19 | 2.54 | 0.0196 |
| [GO:0007596](http://amigo.geneontology.org/amigo/term/GO:0007596) | Blood coagulation | 3 of 173 | 1.76 | 0.0196 |
| **KEGG pathway** | **Description** | **Count in network** | **Strength** | **FDR p-value** |
| hsa04610 | Complement and coagulation cascades | 3 of 82 | 2.08 | 0.00052 |
| **Reactome pathway** | **Description** | **Count in network** | **Strength** | **FDR p-value** |
| HSA-381426 | Regulation of Insulin-like Growth Factor (IGF) transport and uptake by Insulin-like Growth Factor Binding Proteins (IGFBPs) | 5 of 124 | 2.12 | 1.52E-07 |
| HSA-8957275 | Post-translational protein phosphorylation | 4 of 107 | 2.09 | 1.62E-05 |
| HSA-114608 | Platelet degranulation | 3 of 126 | 1.89 | 0.0041 |
| HSA-140877 | Formation of Fibrin Clot (Clotting Cascade) | 2 of 39 | 2.23 | 0.0208 |
| **STRING Cluster** | **Description** | **Count in network** | **Strength** | **FDR p-value** |
| CL:18733 | Mixed, incl. COVID-19, thrombosis and anticoagulation, and Inter-alpha-trypsin inhibitor heavy chain C-terminus | 3 of 21 | 2.67 | 0.00014 |
| CL:18726 | Complement and coagulation cascades, and Protein-lipid complex | 4 of 161 | 1.91 | 0.00014 |
| CL:18737 | Fibrinogen, and Thrombophilia | 2 of 6 | 3.04 | 0.0011 |

**Table S2**: Proteins (genes) with 24 hour rhythm analysed by STRING, pathway outputs

| **GO term** | **Description** | **Count in network** | **Strength** | **FDR p-value** |
| --- | --- | --- | --- | --- |
| GO:0034382 | Chylomicron remnant clearance | 2 of 5 | 3.12 | 0.0021 |
| GO:0043152 | Induction of bacterial agglutination | 2 of 6 | 3.04 | 0.0021 |
| GO:0032489 | Regulation of Cdc42 protein signal transduction | 2 of 8 | 2.91 | 0.0029 |
| GO:0090209 | Negative regulation of triglyceride metabolic process | 2 of 10 | 2.82 | 0.004 |
| GO:0031639 | Plasminogen activation | 2 of 11 | 2.78 | 0.0045 |
| **KEGG pathway** | **Description** | **Count in network** | **Strength** | **FDR p-value** |
| hsa04610 | Complement and coagulation cascades | 4 of 82 | 2.2 | 1.69E-06 |
| hsa04979 | Cholesterol metabolism | 2 of 48 | 2.14 | 0.0158 |
| hsa05150 | Staphylococcus aureus infection | 2 of 86 | 1.88 | 0.0327 |
| hsa04611 | Platelet activation | 2 of 122 | 1.73 | 0.0487 |
| **Reactome pathway** | **Description** | **Count in network** | **Strength** | **FDR p-value** |
| HSA-8964058 | HDL remodeling | 2 of 10 | 2.82 | 0.0116 |
| HSA-8963901 | Chylomicron remodeling | 2 of 10 | 2.82 | 0.0116 |
| HSA-8963888 | Chylomicron assembly | 2 of 10 | 2.82 | 0.0116 |
| HSA-372708 | p130Cas linkage to MAPK signaling for integrins | 2 of 15 | 2.64 | 0.0116 |
| HSA-354194 | GRB2:SOS provides linkage to MAPK signaling for Integrins | 2 of 15 | 2.64 | 0.0116 |
| **STRING Cluster** | **Description** | **Count in network** | **Strength** | **FDR p-value** |
| CL:18726 | Complement and coagulation cascades, and Protein-lipid complex | 6 of 161 | 2.09 | 1.55E-09 |
| CL:18737 | Fibrinogen, and Thrombophilia | 3 of 6 | 3.22 | 1.20E-06 |
| CL:18728 | Complement and coagulation cascades, and Positive regulation of opsonization | 4 of 109 | 2.08 | 9.96E-06 |
| CL:18956 | Lipoprotein particle, and Assembly of active LPL and LIPC lipase complexes | 2 of 46 | 2.15 | 0.0305 |

**Table S3**: List of proteins identified in this work, split rhythmic and non-rhythmic

| **Uniprot ID** | **Protein short name** | **Gene Names** | **24 Hour p-value** | **12 Hour  p-value** |
| --- | --- | --- | --- | --- |
| **Rhythmic proteins** | | | | |
| P25311 | Zinc-alpha-2-glycoprotein | AZGP1 |  | 0.0088 |
| P02671 | Fibrinogen alpha chain | FGA | 0.0255 | 0.0188 |
| P19823 | Inter-alpha-trypsin inhibitor heavy chain H2 | ITIH2 |  | 0.0196 |
| P01042 | Kininogen-1 | KNG1 |  | 0.0203 |
| P02652 | Apolipoprotein A-II | APOA2 |  | 0.0208 |
| P0DOX7 | Immunoglobulin kappa light chain |  |  | 0.0390 |
| P00747 | Plasminogen | PLG | 0.0100 | 0.0394 |
| P01871 | Immunoglobulin heavy constant mu | IGHM |  | 0.0416 |
| P01834 | Immunoglobulin kappa constant | IGKC |  | 0.0493 |
| P02656 | Apolipoprotein C-III | APOC3 | 0.0107 |  |
| P02675 | Fibrinogen beta chain | FGB | 0.0344 |  |
| P08603 | Complement factor H | CFH | 0.0416 |  |
| P02649 | Apolipoprotein E | APOE | 0.0432 |  |
| **Non-rhythmic proteins** | | | | |
| P00450 | Ceruloplasmin | CP |  |  |
| P00738 | Haptoglobin | HP |  |  |
| P01009 | Alpha-1-antitrypsin | SERPINA1 |  |  |
| P01023 | Alpha-2-macroglobulin | A2M |  |  |
| P01024 | Complement C3 | C3 |  |  |
| P01859 | Immunoglobulin heavy constant gamma 2 | IGHG2 |  |  |
| P01860 | Immunoglobulin heavy constant gamma 3 | IGHG3 |  |  |
| P01876 | Immunoglobulin heavy constant alpha 1 | IGHA1 |  |  |
| P02647 | Apolipoprotein A-I | APOA1 |  |  |
| P02679 | Fibrinogen gamma chain | FGG |  |  |
| P02749 | Beta-2-glycoprotein 1 | APOH |  |  |
| P02765 | Alpha-2-HS-glycoprotein | AHSG |  |  |
| P02768 | Albumin | ALB |  |  |
| P02774 | Vitamin D-binding protein | GC |  |  |
| P02787 | Serotransferrin | TF |  |  |
| P02790 | Hemopexin | HPX |  |  |
| P10909 | Clusterin | CLU |  |  |
| P68871 | Hemoglobin subunit beta | HBB |  |  |
| P69905 | Hemoglobin subunit alpha | HBA1 |  |  |
| P01861 | Immunoglobulin heavy constant gamma 4 | IGHG4 |  |  |
| P02763 | Alpha-1-acid glycoprotein 1 | ORM1 |  |  |
| P06727 | Apolipoprotein A-IV | APOA4 |  |  |
| P0DOY2 | Immunoglobulin lambda constant 2 | IGLC2 |  |  |
| P01008 | Antithrombin-III | SERPINC1 |  |  |
| P04196 | Histidine-rich glycoprotein | HRG |  |  |
| P01011 | Alpha-1-antichymotrypsin | SERPINA3 |  |  |
| P02654 | Apolipoprotein C-I | APOC1 |  |  |
| P00734 | Prothrombin | F2 |  |  |
| P0DOX2 | Immunoglobulin alpha-2 heavy chain |  |  |  |
| P00751 | Complement factor B | CFB |  |  |
| P04217 | Alpha-1B-glycoprotein | A1BG |  |  |
| P05155 | Plasma protease C1 inhibitor | SERPING1 |  |  |
| P04004 | Vitronectin | VTN |  |  |
| Q14624 | Inter-alpha-trypsin inhibitor heavy chain H4 | ITIH4 |  |  |
| P06396 | Gelsolin | GSN |  |  |
| P19652 | Alpha-1-acid glycoprotein 2 | ORM2 |  |  |
| P01780 | Immunoglobulin heavy variable 3-7 | IGHV3-7 |  |  |
| P01619 | Immunoglobulin kappa variable 3-20 | IGKV3-20 |  |  |
| B9A064 | Immunoglobulin lambda-like polypeptide 5 | IGLL5 |  |  |
| P05090 | Apolipoprotein D | APOD |  |  |
| P19827 | Inter-alpha-trypsin inhibitor heavy chain H1 | ITIH1 |  |  |
| P01591 | Immunoglobulin J chain | JCHAIN |  |  |
| A0A0B4J1U7 | Immunoglobulin heavy variable 6-1 | IGHV6-1 |  |  |
| P08697 | Alpha-2-antiplasmin | SERPINF2 |  |  |
| A0A0B4J1V0 | Immunoglobulin heavy variable 3-15 | IGHV3-15 |  |  |
| P01019 | Angiotensinogen | AGT |  |  |
| P01615 | Immunoglobulin kappa variable 2D-28 | IGKV2D-28 |  |  |
| P01624 | Immunoglobulin kappa variable 3-15 | IGKV3-15 |  |  |
| P0C0L5 | Complement C4-B | C4B |  |  |
| P02760 | Protein AMBP | AMBP |  |  |
| P0C0L4 | Complement C4-A | C4A |  |  |
| P02747 | Complement C1q subcomponent subunit C | C1QC |  |  |
| P02766 | Transthyretin | TTR PALB |  |  |
| P27169 | Serum paraoxonase/arylesterase 1 | PON1 |  |  |
| P00739 | Haptoglobin-related protein | HPR |  |  |
| P80748 | Immunoglobulin lambda variable 3-21 | IGLV3-21 |  |  |
| P02751 | Fibronectin | FN1 |  |  |
| P0DOX6 | Immunoglobulin mu heavy chain |  |  |  |
| P01857 | Immunoglobulin heavy constant gamma 1 | IGHG1 |  |  |
| P20742 | Pregnancy zone protein | PZP |  |  |
